# Expanding the Substrate Scope of Nitrating Cytochrome P450 TxtE by Active Site Engineering of a Reductase Fusion

**DOI:** 10.1002/cbic.202100145

**Published:** 2021-05-07

**Authors:** Rakesh Saroay, Gheorghe‐Doru Roiban, Lona M. Alkhalaf, Gregory L. Challis

**Affiliations:** ^1^ Department of Chemistry University of Warwick Coventry CV4 7AL UK; ^2^ Synthetic Biochemistry Medicinal Science & Technology GlaxoSmithKline Medicines Research Centre Gunnels Wood Road Stevenage SG1 2NY UK; ^3^ Warwick Integrative Synthetic Biology Centre University of Warwick Coventry CV4 7AL UK; ^4^ Department of Biochemistry and Molecular Biology Monash University Clayton VIC 3800 Australia; ^5^ ARC Centre of Excellence for Innovations in Peptide and Protein Science Monash University Clayton VIC 3800 Australia

**Keywords:** Biocatalysis, Indole, Nitration, Protein Engineering

## Abstract

Aromatic nitration reactions are a cornerstone of organic chemistry, but are challenging to scale due to corrosive reagents and elevated temperatures. The cytochrome P450 TxtE nitrates the indole 4‐position of l‐tryptophan at room temperature using NO, O_2_ and NADPH, and has potential to be developed into a useful aromatic nitration biocatalyst. However, its narrow substrate scope (requiring both the α‐amino acid and indole functionalities) have hindered this. Screening of an R59 mutant library of a TxtE‐reductase fusion protein identified a variant (R59C) that nitrates tryptamine, which is not accepted by native TxtE. This variant exhibits a broader substrate scope than the wild type enzyme and is able to nitrate a range of tryptamine analogues, with significant alterations to the aromatic and aminoethyl moieties.

## Graphical abstract

**Directed evolution of aromatic nitration biocatalysts**: Screening variants of a self‐sufficient reductase fusion of the indole‐nitrating cytochrome P450 TxtE led to the discovery of an active site mutant that nitrates tryptamine and a range of tryptamine analogues with alterations to the aminoethyl group and/or indole.

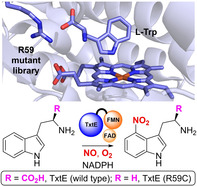

Aromatic nitration is an important industrial process used in the preparation of dyes, pesticides, food additives and pharmaceuticals.^[1]^ Large scale aromatic nitration is currently achieved using nitric acid and sulfuric acid. However, this method has several drawbacks, including a lack of selectivity and a high environmental impact.^[2]^ Alternative, more environmentally benign methods for aromatic nitration thus need to be developed.

In 2012, the cytochrome P450 (CYP) TxtE was shown to catalyse regiospecific nitration of l‐tryptophan at the indole 4‐position during the biosynthesis of thaxtomin A, a phytotoxin produced by *Streptomyces scabies* and related plant pathogens.^[3]^ TxtE employs a combination of O_2_, NO and an electron from NADPH (transferred via ferredoxin (Fd) and ferredoxin reductase (Fr) redox partner proteins) to effect the nitration reaction. In *S. scabies* TxtD, a nitric oxide synthase, supplies NO to TxtE by converting l‐arginine to l‐citrulline using O_2_ and NADPH.[^3^, ^4^] *In vitro* TxtD can be replaced with the sodium salt of 2‐(*N*,*N*‐diethylamino)‐diazenolate‐2‐oxide (DEANO), which functions as an NO donor in aqueous solution.

TxtE has been shown to accept several tryptophan analogues with additional substituents appended to the carbocycle or α‐carbon as substrates, although product yields were generally low (Figure 1).^[5]^ However, analogues containing other heterocycles are not tolerated, and both the carboxyl and amino groups in tryptophan and the majority of accepted analogues appear to be required for productive binding to the active site (Figure 1).^[5]^ Indeed, tryptophan analogues that are not accepted as substrates, such as tryptamine, have been shown by UV/Vis spectroscopy to ligate the heme iron, preventing dioxygen binding.^[6]^ The requirement for an l‐configured α‐amino acid is a significant impediment to developing TxtE into a useful biocatalyst, due to the complexities associated with enantioselective substrate synthesis. Moreover, the carboxyl and amino groups are both reactive and awkward to manipulate, necessitating multistep protection and functional group interconversion strategies to utilize products of wild type TxtE‐catalysed nitration in complex molecule synthesis. Whilst a TxtE variant that nitrates l‐Trp at the 5‐position has been identified, alteration of the enzyme's substrate specificity has yet to be reported.^[7]^

**Figure 1:**
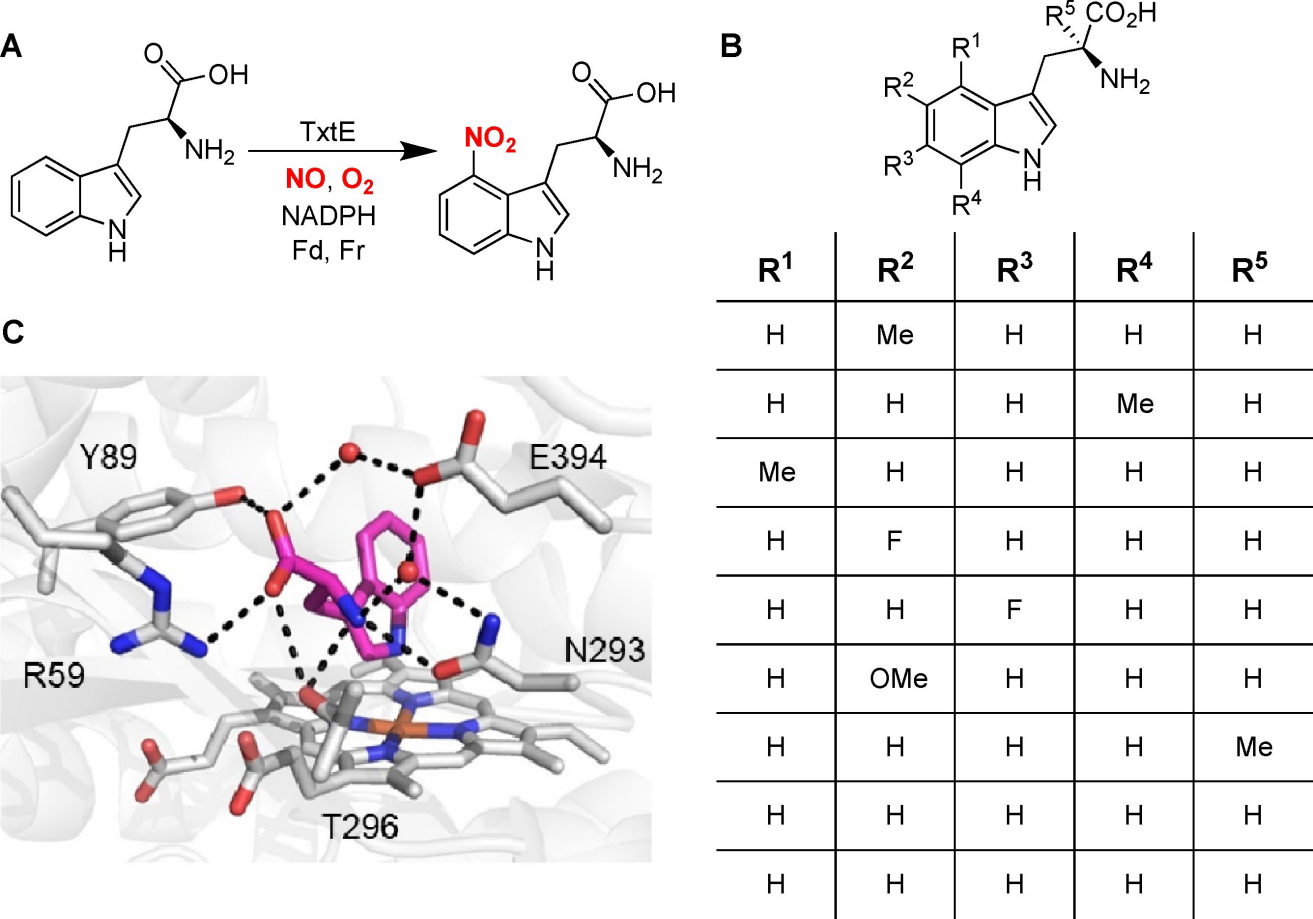
(A) Reaction catalysed by TxtE in thaxtomin biosynthesis. (B) Tryptophan analogues accepted by wildtype TxtE (C) X‐ray crystal structure of TxtE with l‐tryptophan bound (PDB accession: 4TPO), highlighting hydrogen bonds between the amino/carboxyl groups of the substrate and the side chains of residues lining the active site including R59 which was mutated in this study. Red spheres represent ordered water molecules.

Here we describe the development of high‐throughput methodology for rapid screening of TxtE variants, resulting in the identification of an active site mutant (R59C) that is able to nitrate tryptamine and several analogues with a range of modifications to the indole and/or aminoethyl groups. The discovery of TxtE variants able to accept structurally‐simplified and more diverse substrate analogues is an important first step towards the development of useful nitration biocatalysts.

To identify TxtE variants capable of accepting a wider range of substrates, it was necessary to develop a high‐throughput screen for enzymatic activity. Thus, we initially sought to eliminate the dependence of TxtE on exogenous Fd and Fr (which are expensive and unstable) for electron transfer from NADPH by fusing it with a suitable reductase. The reductase domains from naturally occurring self‐sufficient CYPs, such as P450_RhF_ and P450_BM3_, which are [2Fe‐2S]/FMN and FAD/FMN‐dependent, respectively, have previously been fused to several CYPs resulting in self‐sufficient fusion proteins.[^8^, ^9^] We therefore initially created a TxtE‐RhF reductase fusion, which was found to be capable of self‐sufficient nitration. However, turnover was low, prompting us to pursue a TxtE‐BM3 reductase (BM3R) fusion instead. A gene encoding a codon optimised His_6_‐TxtE‐BM3R fusion (to promote high level expression in *E. coli*) that included the full length heme‐reductase linker (27 aa) from CYP102A1 was designed, synthesised and cloned into a suitable expression vector (Figures S1 and S2). The resulting protein was overproduced in *E. coli* and purified to homogeneity (Figure S2). Incubation of the purified protein with l‐tryptophan, NADPH and DEANO resulted in production of 4‐nitrotryptophan (Figure S3). The total turnover number (TTN) for the fusion protein was 450, which is similar to that for our previously reported His_6_‐TxtE construct,^[3]^ when spinach Fd and Fr are employed as redox partners (TTN=580). We then developed and optimised a cell lysate‐based assay system that employs glucose and glucose dehydrogenase to regenerate NADPH *in situ* from NADP^+^.

With this cell lysate‐based assay system in hand, we turned our attention to the creation of TxtE variants with altered substrate specificity. The X‐ray crystal structure of TxtE with l‐tryptophan bound shows an extensive network of hydrogen bonds and electrostatic interactions between the amino and carboxyl groups of the substrate and the side chains of the R59, Y89, N293, T296 and E394 active site residues, involving two ordered water molecules (Figure 1C).^[5]^ Among these, a direct electrostatic interaction between the guanidinium group of R59 and the carboxyl group of the substrate was hypothesized to be a key selectivity determinant for substrates containing a carboxylic acid. Using saturation mutagenesis, we created a library of His_6_‐TxtE‐BM3R mutants in which R59 is substituted by other proteinogenic amino acids. Randomisation was achieved in a single step using the Q5‐mutagenesis protocol in conjunction with the 22c‐trick.^[10]^ Colonies were arrayed into a 96‐well plate and grown, and expression of the variant genes was induced using IPTG. The ability of cell lysates to nitrate tryptamine **1** was analysed using LC‐MS. The plasmids from wells displaying activity were isolated and their inserts were sequenced, identifying mutants in which R59 has been replaced with S or C (Figure S4).

The R59C mutant appeared to be slightly more active than the R59S mutant and was thus selected for further analysis. Purified His_6_‐TxtE‐BMR3(R59C) was incubated with tryptamine **1**, NADPH and DEANO. LC‐MS analysis of the reaction mixture revealed a major and a minor product with *m/z* values corresponding to [M+H]^+^ for nitrated tryptamine (Figure 2). The molecular formulae of these products were confirmed using UHPLC‐ESI‐Q‐TOF‐MS (Table S5). However, purification of the major product in sufficient quantities for NMR spectroscopic analysis proved challenging. While our work was in progress, Zuo *et al*. reported that TxtE‐BM3R fusions in which the heme‐reductase linker is shortened from 27aa to 14aa have increased activity.^[11]^ We therefore created a His_6_‐TxtE‐BMR3(R59C)Δ construct with a similar shortened linker (Figure S1), which enabled purification of the major product using semi‐preparative HPLC and subsequent ^1^H NMR spectroscopic analysis. The pattern of signals due to the aromatic protons was consistent with nitration at the indole 4 or 7‐position and comparison with a synthetic standard of 4‐nitrotryptamine confirmed this is the major product of the reaction (Figure S5). The minor product could not be purified in sufficient quantity to characterize by ^1^H NMR spectroscopy, but is presumably an indole nitration regioisomer.

**Figure 2:**
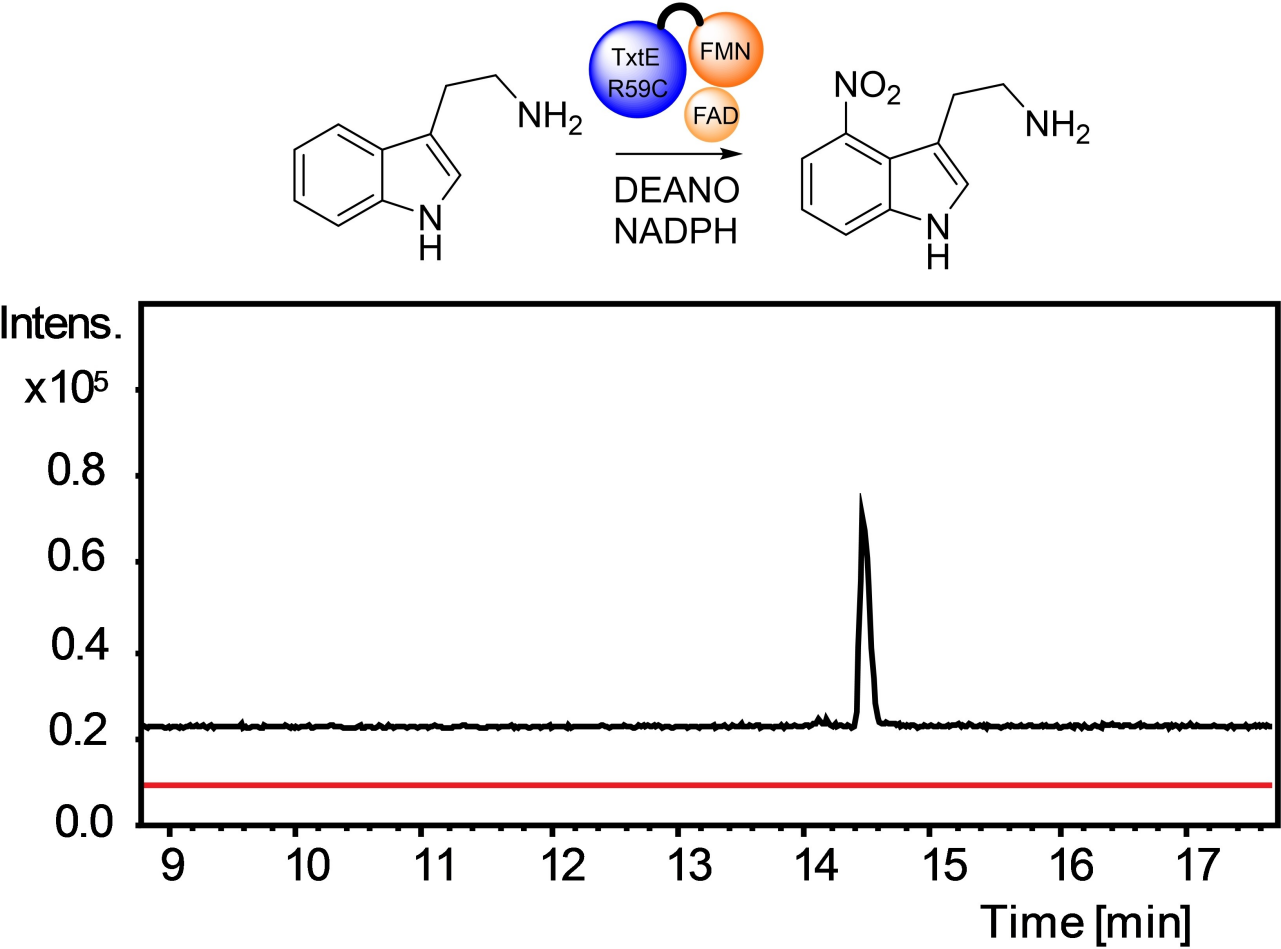
Nitration of tryptamine catalysed by TxtE‐BMR3(R59C). Extracted ion chromatograms at *m/z*=206.924±0.005 (corresponding to [M+H]^+^ for nitrotryptamine) from LC‐MS analyses of tryptamine incubated with DEANO, NADPH, and His_6_‐TxtE‐BM3R(R59C) (black) or heat denatured enzyme (red).

Consistent with our hypothesis that R59 plays an important role in the recognition of substrates containing carboxylic acids, the R59C mutant was much less active towards l‐tryptophan than the unmodified reductase fusion (Figure S6). UV‐Vis difference spectra indicated that the mutant protein has a lower affinity for l‐tryptophan (Figure S6) and a concentration of 5 mM was insufficient to saturate its active site (*K*_d_=84±2 μM for binding of l‐tryptophan to His_6_‐TxtE‐BMR3 (Figure S3)).

A type II difference spectrum is observed for binding of tryptamine to wild type TxtE,^[6]^ indicative of heme iron coordination, which explains why it is not a substrate. Intriguingly, a similar spectrum is observed for binding of tryptamine to His_6_‐TxtE‐BMR3(R59C) (Figure S7). Despite this, it appears that the R59C mutation permits some tryptamine binding in an alternative mode, allowing nitration to occur.

To further explore the substrate scope of His_6_‐TxtE‐BM3R(R59C)Δ, we initially tested three analogues of tryptamine (**2**, **3**, and **4**) with additional functional groups on the indole (Fig. 3), for which the corresponding l‐tryptophan analogues have previously been reported to be substrates of wild type TxtE (Figure 1).^[5]^ LC‐MS analyses showed all of these are converted to nitrated products (Figure S8 and Table S5). While **3** and **4** yielded one main product, the nitration of **2** was less selective, resulting in a ∼1 : 1 mixture of two regioisomers (Figure S8).

Encouraged by these results, we examined more diverse tryptamine analogues with structures that do not correspond to substrate analogues accepted by wild type TxtE (Figure 3). Tryptophanol **5** yielded a single nitrated product (Figure S8 and Table S5). On other hand, indole‐3‐propionic acid **6**, tryptophol **7**, acetamide **8** and hydrazide **9** could not be nitrated by the engineered enzyme, indicating that the nature and location of the heteroatom(s) in the substituent attached to the indole 3‐position plays an important role in substrate recognition. We also investigated the tricyclic analogues tryptoline **10** and 2‐hydroxycarbazole **11**. While the former gave a ∼1 : 1 mixture of two regioisomeric nitration products (Figure S8 and Table S5), the latter was not turned over.

**Figure 3:**
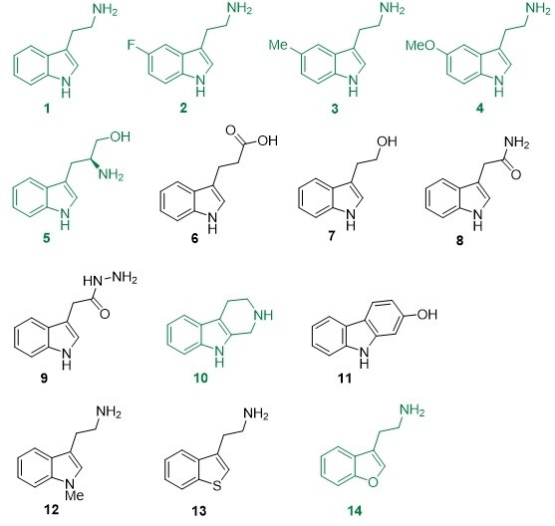
Analogues of tryptamine investigated as substrates of His_6_‐TxtE‐BMR3(R59C)Δ. Compounds in green yielded nitrated product(s), whereas those in black did not.

We recently proposed a catalytic mechanism for TxtE based on a combination of stopped‐flow kinetics experiments and density functional theory calculations (Figure 4).^[12]^ This involves formation of a ferric superoxide complex that couples with NO to form a ferric peroxynitrite intermediate. Homolytic cleavage of the O−O bond in this intermediate affords an Fe(IV)=O complex and NO_2_, which adds to the indole 4‐position of l‐tryptophan. Abstraction of the *ipso*‐hydrogen atom by the Fe(IV)=O complex yields the nitrated product. However, *N*‐methyl‐l‐tryptophan has been shown to bind to the active site of TxtE, but does not undergo nitration,^[5]^ suggesting that abstraction of hydrogen from the indole N−H may precede the addition of NO_2_ to the aromatic ring. This would be in accord with the mechanism for nitration of 3‐substitued indoles by NO_2_ in free solution, which involves hydrogen abstraction from the indole nitrogen atom.^[13]^

**Figure 4:**
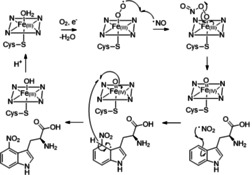
Proposed catalytic mechanism of TxtE. Substrate binding, heme reduction, loss of the water ligand and dioxygen binding results in a ferric‐superoxide complex, as in hydroxylating CYPs. Reaction with NO forms a ferric‐peroxynitrite intermediate that homolyses to form an Fe(IV)=O complex and nitrogen dioxide, which adds to the π system of the substrate. Abstraction of the *ipso*‐hydrogen atom by the Fe(IV)=O complex affords the product and a ferric hydroxide complex, which undergoes protonation to complete the catalytic cycle.

To further probe the catalytic mechanism of TxtE, we tested tryptamine analogues with modifications to the heteroatom of the indole (Figure 3). Neither N‐methyltryptamine **12**, nor the benzothiophene analogue **13** gave nitrated products, whereas the benzofuran analogue **14** afforded a single nitrated derivative (Figure S9). The observation that N‐methyltryptamine **12** is not nitrated by His_6_‐TxtE‐BM3R(R59C)Δ is consistent with the inability of wild type TxtE to nitrate l‐tryptophan.^[5]^ However, the fact that benzofuran **14** undergoes nitration provides further evidence that the enzyme is able to catalyse the direct attack of NO_2_ on the HOMO of moderately electron rich aromatic species. The failure of benzothiophene **13** to undergo nitration probably reflects the relative inability of the sulphur lone pair, compared with the nitrogen and oxygen lone pairs, to stabilise the radical resulting from addition of NO_2_ to the aromatic ring, which is a consequence of poorer orbital overlap.^[14]^

In conclusion, we have created an expression construct for a TxtE‐BM3R fusion that enables direct high throughput screening of *E. coli* cell lysates for enzyme variants with altered properties. This construct was exploited to create and screen a saturation mutagenesis library targeting R59, which is hypothesized to be a key determinant of substrate specificity. R59C and R59S mutants were identified that catalyse efficient nitration of tryptamine, a substrate that is not accepted by the native enzyme. The R59C mutant was also able to nitrate structurally diverse tryptamine analogues with modifications to both aromatic rings and the aminoethyl substituent. This demonstrates the applicability of protein engineering approaches to the creation of TxtE variants with altered and expanded substrate tolerance, highlighting the potential for CYPs to be developed into useful nitration biocatalysts.

## Conflict of interest

G.L.C. is a non‐executive director of Erebagen Ltd.

## Supporting information

As a service to our authors and readers, this journal provides supporting information supplied by the authors. Such materials are peer reviewed and may be re‐organized for online delivery, but are not copy‐edited or typeset. Technical support issues arising from supporting information (other than missing files) should be addressed to the authors.

Supplementary
