# Supplementary material for: Expanding the Substrate Scope of Nitrating Cytochrome P450 TxtE by Active Site Engineering of a Reductase Fusion
Source: Chembiochem. 2021 May 7;22(13):2262–5. doi: 10.1002/cbic.202100145 (PMC8359946; doi:10.1002/cbic.202100145)
Supplement: Supplementary file 1 — Supplementary [file CBIC-22-2262-s001.pdf]

ChemBioChem

Supporting Information

Expanding the Substrate Scope of Nitrating Cytochrome P450 TxtE by Active Site Engineering of a Reductase Fusion

Rakesh Saroay, Gheorghe-Doru Roiban, Lona M. Alkhalaf,* and Gregory L. Challis*

Contents

1. General 1
1.1 Chemicals and reagents 1
1.2 Instrumentation 2
1.3 Plasmids, bacterial strains and enzymes 3
1.4 Biological reagents and media 3
2. Biological procedures 3
2.1 General procedure for protein expression 3
2.2 Protein analysis and characterisation 4
2.3 Generation of TxtE-BM3R mutant library 4
2.4 High-throughput screening and analysis of the His_6-TxtE-BM3R mutant libraries 5
2.5 In vitro activity assays of His_6-TxtE-BMR3(R59C) with tryptamine and analogues 6
2.6 Scale-up of the tryptamine nitration using His_6-TxtE-BMR3(R59C)Δ 6
2.7 Qualitative binding assay – determining protein-substrate binding type 7
2.8 Determination of substrate dissociation constants 7
3. Chemical synthesis 7
3.1 Synthesis of 4-nitrotryptamine 7
3.1.1 Synthesis of 4-nitro-L-tryptophan 7
3.1.2 Synthesis of 4-nitrotyptamine 8
3.2 Synthesis of 2-(benzo[b]thiophen-3-yl)ethanamine 8
4. Supplementary figures 9
5. Supplementary tables 15
6. References 16

1. General

1.1 Chemicals and reagents

All chemicals and reagents were purchased from Sigma-Aldrich, Fisher Scientific, Alfa Aesar, VWR International, Acros Organics or Fluorochem and were used as supplied unless stated otherwise. Solvents were sourced from either Sigma-Aldrich or Fisher Scientific. Thin-layer chromatography was performed on silica-coated aluminium from Merck (TLC silica gel 60 F_{254}) and visualised using UV fluorescence and/or ninhydrin stain. Silica column chromatography was performed using silicon

dioxide from Sigma-Aldrich (40-63 μm, 60 Å pore size). NMR solvents were purchased from Sigma-Aldrich or Cambridge Isotope Laboratories.

1.2 Instrumentation

NMR spectra were obtained on either a Bruker Avance III 400 MHz spectrometer at 400 MHz (^{1}H) and 100 MHz (^{13}C), or a Bruker Avance III 500 MHz spectrometer equipped with a DCH cryoprobe for at 500 MHz (^{1}H) and 125 MHz (^{13}C). Spectra were recorded at 25 ° C in D_2O, DCl in D_2O or MeOD. Chemical shifts are reported as δ values in ppm relative to the residual HOD (4.80 ppm (^{1}H)) or CH_3OD (3.34 ppm (1H) and 49.8 ppm (^{13}C)) signals. Coupling constants, *J*, are reported in Hz, with multiplicities quoted as singlet (s), doublet (d), triplet (t), quartet (q), doublet of doublets (dd), doublet of triplets (dt), or multiplet (m). Low resolution mass spectra were measured on an Agilent 6130 ESI-Quadrupole instrument. Low resolution LC-MS analyses were performed on a Bruker AmaZon ESI spectrometer connected to an Agilent 1200 HPLC instrument fitted with an Agilent Eclipse Plus C18 column (5μm, 4.6 x 150 mm). High resolution UHPLC-MS analyses were performed on a Bruker MaXis Impact ESI-Q-TOF spectrometer connected to a Dionex 3000 RS UHPLC instrument fitted with an Agilent Zorbax Eclipse Plus C18 column (100 x 2.1 mm, 1.8 μm). HRMS, 500 MHz ^{1}H NMR and 125 MHz ^{13}C NMR spectra were obtained by the departmental Mass Spectrometry and NMR services run by Dr Lijiang Song and Dr Ivan Prokes, respectively. UV-visible spectra were recorded on a PerkinElmer Lambda 35 instrument at 25 °C. LC-MS analysis of 96-well plates were carried out on a Waters ZQ instrument using an Acquity UPLC CSH C18 column (50mm x 2.1mm i.d. 1.7μm) column and a flow rate of 1.0 ml min^{-1}. Mass-directed auto purification (MDAP) was carried out on a Waters QDA instrument using an Xselect CSH C18 column (150mm x 30mm i.d. 5μm) column a flow rate of 40 ml min^{-1}.

Cells pellets were lysed using a microfluidiser M110Y from analytikLtd. Lyophilization was performed using a BPS VirTris SP Scientific Advantage Pro lyophilizer. Hight throughtput screening reactions were carried out in Costar deep-well 96-well plates, and UPLC analysis performed in shallow well polypropylene plates (Nunc 96). A BioMex FX liquid handling robot (Beckman Coulter, Fullerton, CA) was used for assay setup.

Polymerase chain reactions were performed in either a Bio-Rad T100 thermocycler or an Eppendorf Mastercycler Gradient. DNA agarose gel electrophoresis was performed in a Bio-Rad Wide Mini-Sub Cell GT tank connected to a Bio-Rad Basic PowerPac power supply. PCR products were visualised on UVP UV transilluminating imaging system. SDS-PAGE analyses were conducted in a Bio-Rad Mini-PROTEAN Tetra Vertical Electrophoresis Cell connected to a Bio-Rad Basic PowerPac power supply. DNA and protein concentrations were measured using a Thermo Scientific NanoDrop Lite spectrometer. Cell growth and protein expression was conducted in New Brunswick Scientific Innova44

incubators and cells were lysed using a Constant Systems cell disruptor. Cell optical density was measured using a Thermo BioMate3 spectrometer at 600 nm.

1.3 Plasmids, bacterial strains and enzymes

The construction of pSB13, containing the gene for *txtE* from *Streptomyces turgidiscabies* Car8 in pET-151, has been described previously.[1] The gene encoding TxtE-BM3R, codon-optimised for *E. coli*, cloned as a His_6 translational fusion into pET28a(+) was purchased from GenScript, NJ, USA. Competent cells were were either Invitrogen One Shot TOP10 and BL21 Star (DE3) or New England Biolabs DH5α and BL21(DE3). Restriction enzymes were purchased from either ThermoFisher Scientific or New England Biolabs.

1.4 Biological reagents and media

Miller's Luria-Bertani (LB) media was purchased from Fisher Scientific and prepared to 25 g/L in deionised water. Solid LB-agar media was prepared using 25 g/L LB broth and 15 g/L bacto agar in deionised water. Terrific broth (TB) was purchased from VWR and prepared by adding 50 g of powder to 990 mL deionised water and 10 mL glycerol prior to autoclaving. Ampicillin and kanamycin stock solutions were prepared to 100 mg/mL and 50 mg/L, respectively, and filter sterilised prior to being stored at -20˚C until use. Isopropyl β-D-1-thiogalactopyranoside (IPTG) purchased from VWR was prepared as a 0.5 M stock solution in deionised water, filter sterilised and stored at -20˚C until use.

2. Biological procedures

2.1 General procedure for protein expression

The general procedure for the expression of His_6-TxtE-BM3R and His_6-TxtE-BMR3(R59C) is as follows: the construct was used to transform *E. coli* BL21Star (DE3). A single transformant from antibiotic-supplemented LB-agar plates was used to inoculate LB medium (10 mL) supplemented with kanamycin (50 µg/mL) and the resulting culture was incubated at 30 ºC overnight with shaking at 220 rpm. This overnight culture (5-10 mL) was used to inoculate LB or TB medium (1 L) supplemented with antibiotic and was incubated at 37 ºC with shaking at 220 rpm until the optical density at 600 nm reached 0.6-0.8. The culture was allowed to cool and isopropyl-β-D-thiogalactopyranoside (IPTG) (0.1 mM), 5-aminolevulinic acid hydrochloride (5-ALA) (1 mM), iron (III) chloride (5 mg/L) and thiamine (1 mM) were added. The culture was incubated overnight at 15 ºC with shaking at 220 rpm. Cells from the overnight incubation were harvested by centrifugation (4000 x *g*, 30 min), re-suspended in 10 mL Buffer A (table S1) and phenylmethylsulphonyl fluoride (PMSF, 1 mM final conc.) was added. The cells were lysed and cell debris was pelleted by centrifugation (11,000 x *g*, 30 min). The supernatant was passed through a 0.2 µm syringe filter and applied to a 1 mL HiTrap™ HP affinity column (Nickel

Sepharose High Performance, GE Healthcare) equilibrated with Buffer A. Unbound proteins were eluted with Buffer A (10 mL) and His_6-TxtE-BM3R and the R59C mutant were eluted using 5, 3, 3, and 3 mL of buffers B1-B4, respectively. The orange fractions were pooled and concentrated to ~ 300 µL (Ultra centrifugal device 50 kDa membrane) before being diluted with Buffer C and concentrated again. This concentration procedure was repeated twice more before the purity of the collected fractions was analysed by SDS-PAGE (10%), and the purified protein was flash frozen in aliquots using liquid nitrogen for storage at -80 ºC until further use.

2.2 Protein analysis and characterisation

SDS-PAGE analysis of purified proteins was conducted using a 10% acrylamide/bis-acrylamide gel. The loading gel was allowed to set for 30 minutes prior to the addition of the stacking gel plus comb. This was set for at least 30 minutes before conducting the analysis. The electrophoresis tank was filled with running buffer (2 M glycine, 1% SDS, 250 mM Tris-HCl, pH 8.0) and the set gel was appropriately placed inside. Protein samples (10-20 µL) were prepared firstly by the addition of loading dye (2-4 µL, 5X stock contains 250 mM Tris·HCl, pH 6.8, 10% SDS, 30% (v/v) Glycerol, 10 mM DTT, 0.05% (w/v) Bromophenol Blue). The samples were heated to 80 ºC for 5 minutes before being applied to the SDS-PAGE gel lane alongside protein ladder (3 µL, Thermo Scientific Page Ruler Plus Prestained). The gel was run at 200 V for 35 minutes and visualised using InstantBlue Protein Stain (Expedeon).
An estimate of the percentage of active heme-containing protein in the solution was calculated by UV-visible spectroscopy. A solution of the purified protein (10-20 µL) was added to a cuvette containing Tris-HCl (25 mM, pH 8.0) and a spectrum was measured between 200 and 500 nm using a blank cuvette containing Tris-HCl (25 mM, pH 8.0) as a reference. The ratio (A_{420nm}/A_{280nm}) x 100, was used as an estimate for the percentage of total incorporated heme and was used to calculate an accurate yield of active cytochrome P450 in the protein solution.
Total protein concentration was calculated using a Nanodrop Lite instrument with the absorbance units set to 1 mg/mL. Using the Beer-Lambert law, $A = \varepsilon lc$, where A is the measured absorbance, l is the path length (1 cm), and ε is the predicted extinction coefficients. The protein concentration, c, was calculated for each protein prior to use.

2.3 Generation of TxtE-BM3R mutant library

The codon for R59 in the His_6-TxtE-BM3R construct was randomised in one step using the Q5-site directed mutagenesis protocol (New England Biolabs). The three mutagenic forward primers encoding NDT, VHG and TGG at the site of saturation were mixed in a ratio of 12:9:1, respectively, to a final concentration of 10 µM. The PCR reaction mixture (50 µL final volume) contained 25 µL Q5 hot start polymerase, 2.5 µL forward primer mixture (10 µM, GACCGCGGAC**NDT**GGTACCGAGG, GACCGCGGAC**VHG**GGTACCGAGG, GACCGCGGAC**TGG**GGTACCGAGG), 2.5 µL reverse

primer (10 µM, AGACGCGGGTCCTTCAGACCCG), 2 µL template DNA (10 ng/µL) and 18 µL nuclease-free water. The PCR reaction mixture (50 µL) was split into 4 x 12.5 µL reactions accounting for the different annealing temperatures required (72.0, 70.2, 65.4 and 61.0 ºC). The PCR program consisted of an initial denaturation step at 98 ºC for 30 s, followed by 25 cycles of 98 ºC for 10 s, annealing at the given temperatures for 30 s, elongation at 72 ºC for 4 min 30 s, followed by a final elongation step at 72 ºC for 2 min. The PCR reaction mixtures were combined and 1 µL was added to 5 µL of KLD reaction buffer, 1 µL of 10X KLD enzyme mix and 3 µL nuclease-free water. The mixture was incubated for 5 min at room temperature before being used to transform 5-alpha competent *E. coli* (New England Biolabs). The next day the transformants were rinsed from the agar plate (50 µg/mL kanamycin) using 1 mL LB broth and plasmid DNA was extracted using a DNA miniprep kit (Qiagen). Randomisation at the site of saturation was confirmed for each library by DNA sequencing.

2.4 High-throughput screening and analysis of the His_6-TxtE-BM3R mutant libraries

The site-randomised DNA mixture was used to transform *E. coli* BL21 (DE3) competent cells (Invitrogen). For each individual well in columns 1-11 of a 96-deep well plate, 88 colonies from each transformation were used to inoculate 100 µL LB broth containing kanamycin (50 µg/mL). In column 12, 3 wells containing the culture broth were inoculated with *E. coli* cells transformed with the His_6-TxtE-BM3R expression construct, 3 wells were inoculated with *E. coli* cells transformed with pET28a(+) and 2 wells were left empty for buffer control reactions. Each plate was incubated at 30 ºC overnight with shaking at 220 rpm. The next day 10 µL of the culture in each well was transferred to the corresponding well in a 96-deep well plate containing 1 mL Overnight Express Autoinduction media (Novagen) containing 50 µg/mL kanamycin. The plates were sealed and incubated at 37 ºC with shaking at 800 rpm. Once the cultures had reached an OD_{600nm} = 1.0-1.5, the temperature was lowered to 20 ºC and the plates were incubated for a further 20 h. The plates were centrifuged (4000 x *g*, 10 min), the medium was discarded and the plates containing cell pellets were stored at -80 ºC until required for reaction screening.

The 96-deep well plates containing the cell pellets for the library were removed from the freezer and thawed at room temperature for 30 min. Lysis buffer (300 µL) containing 1 mg/mL lysozyme, 1mg/mL polymixin B and 10 unit/mL benzonase nuclease in 25 mM Tris-HCl, pH 8.0 was added to each well and the plate was incubated at 20 ºC for 2-3 h with shaking at 800 rpm. When lysis was complete the plate was centrifuged (4000 x *g*, 10 min) to remove cell debris and 240 µL of each well solution was transferred to a separate well in a fresh 96-well plate, each containing 330 µL Tris-HCl, pH 8.0, with 1.2 mM tryptamine, 0.1 mM $NADP^+$, 3.5 mM glucose and 0.5 units glucose dehydrogenase. The reaction was started by the addition of 2-(*N*,*N*-Diethylamino)-diazenolate 2-oxide sodium salt hydrate (DEANO) to a final concentration of 2.4 mM. The plate was sealed and incubated at 25 ºC with shaking at 800 rpm for 20 h. The reaction was terminated by the addition of 1 mL methanol and the plate was

shaken at 800 rpm and 20 ºC for 10 min before being centrifuged (4000 x *g*). 200 µL of the supernatant was transferred to a 96-shallow well plate for analysis by LC-MS (elution profile is given in Table S2).

2.5 In vitro activity assays of His_6-TxtE-BMR3(R59C) with tryptamine and analogues

The ability of TxtE-BM3R_R59C to nitrate several tryptamine analogues was tested by preparing the following reactions in 2 mL Eppendorf tubes: 10 µM enzyme in 25 mM Tris-HCl, pH 8.0, containing 2.0 mM substrate **1-14** or L-Trp, 2.4 mM NADPH and 4.0 mM DEANO in a final volume of 100 µL. The reaction was incubated at 20 ºC for 3 h, then quenched using 100 µL methanol. The mixture was filtered using 0.2 µM microspin filter tubes (Corning) and analysed by LC-MS using the elution profile given in Table S3. Following initial LC-MS analysis the molecular formulae of nitrated **1-5**, **10** and L-tryptophan were confirmed by UHPLC-ESI-Q-ToF MS analysis.

2.6 Scale-up of the tryptamine nitration using His_6-TxtE-BMR3(R59C)Δ

TxtE-BM3RΔ was prepared using the Q5-site directed mutagenesis kit: The linker region between TxtE(R59C) and the BM3 reductase domain was shortened from 29 residues to 14 residues by deletion of 45 bases using the forward primer 5'- ACCGAACAGAGCGCGAAGAAAG-3' and reverse primer 5'ACGCAGGCTCAGCGGCAG-3' (Figure S1). An annealing temperature of 71ºC was used.

A single transformant harbouring plasmid, pET28a-(+)-*txtE-bm3rΔ*, from antibiotic-supplemented LB-agar plates was used to inoculate LB medium (20 mL) supplemented with kanamycin (50 µg/mL). The culture was incubated at 30 ºC overnight with shaking at 220 rpm. 5 mL of this overnight culture was used to inoculate 4 x 1 L of TB medium supplemented with antibiotic and the resulting culture was incubated at 37 ºC with shaking at 220 rpm until the optical density at 600 nm reached 0.6-0.8. The cultures were allowed to cool and isopropyl-β-D-thiogalactopyranoside (IPTG) (0.1 mM), 5-aminolevulinic acid hydrochloride (5-ALA) (1 mM), iron (III) chloride (5 mg/L) and thiamine (1 mM) were added. The cultures were incubated overnight at 18 ºC with shaking at 220 rpm and the cells were harvested by centrifugation (4000 x *g*, 30 min). The pellets were re-suspended in 30 mL Buffer A (Table S1) and phenylmethylsulphonyl fluoride (PMSF, 1 mM final conc.) was added. The cells were lysed and cell debris was pelleted by centrifugation (11,000 x *g*, 30 min). The supernatant was passed through a 0.2 µm syringe filter and applied to a 5 mL HiTrap™ HP affinity column (Nickel Sepharose High Performance, GE Healthcare) equilibrated with Buffer A. Unbound proteins were eluted with Buffer A (30 mL) and His_6-TxtE-BM3R(R59C)Δ was eluted using 15, 10, 10, and 10 mL of buffers B1-B4, respectively. The orange fractions were pooled and concentrated to 1 mL (Ultra centrifugal device 50 kDa membrane) before being diluted with 20 mL Reaction Buffer (25 mM Tris-HCl, pH 8.0 and 100 mM NaCl) and concentrated again. Concentration was repeated twice more to ensure complete removal of imidazole from the protein solution. The His_6-TxtE-BM3R(R59C)Δ protein solution (1 mL, 48

mg/mL) was diluted with a further 12 mL Reaction Buffer (RB) in a 50 mL falcon tube, and tryptamine (0.6 mL, 50 mM in 10 % DMSO in RB) and NADPH (0.72 mL, 50 mM in RB) were added. The mixture was stirred at 20 ºC for 5 min, DEANO (0.6 mL, 0.1 M in 10 mM NaOH) was added to start the reaction and the tube was sealed. After 20 h the reaction was quenched with 10 % HCl in MeOH (15 mL), stirred for 30 min and filtered to remove precipitated proteins. The filtrate was concentrated *in vacuo* and freeze-dried to remove the remaining liquid. The residue was dissolved in 2 mL H_2O and purified by HPLC (Table S4).

2.7 Qualitative binding assay – determining protein-substrate binding type

The protein was added to a cuvette containing Tris-HCl (25 mM, pH 8.0) to a concentration of 8 µM. A reference spectrum was recorded between 300 and 500 nm to provide a reference. L-tryptopan or tryptamine were to a final concentration of 2 mM, the spectrum was re-recorded and the reference spectrum was subtracted to give the difference spectrum.

2.8 Determination of substrate dissociation constants

An 8 µM solution of the protein was prepared in Tris-HCl (25 mM, pH 8.0). A solution of the substrate (0.5-1.0 µL titrations, 10-50 mM stock) was added stepwise to the cuvette (final concentration 0-10 mM) and a spectrum was recorded after each addition. A corresponding reference spectrum for each measurement was recorded and subtracted from the measured spectrum. This procedure was repeated until the total volume of substrate solution added to the cuvette was no greater than 5% of the volume of the protein solution. The experiment was conducted in least duplicate for each substrate tested.
The absorbance at λ_{min} was subtracted from the absorbance at λ_{max} and plotted against the concentration of substrate. The data were fitted to the Michaelis-Menten equation using OriginPro 9.1, and the dissociation constants were calculated from the resulting binding curves.

3. Chemical synthesis

3.1 Synthesis of 4-nitrotryptamine

3.1.1 Synthesis of 4-nitro-L-tryptophan

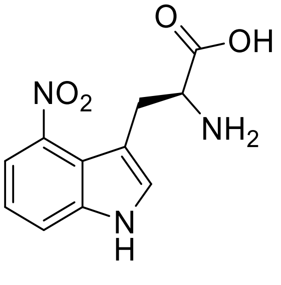

4-Nitro-L-tryptophan was synthesised from 4-nitroindole according to the procedure detailed by Romney et al.[2] ^{1}H NMR (400 MHz, CD_3CN/HCl) δ: 3.28 (1H, dd, J = 9.5, 14.5 Hz), 3.79 (1H, dd, J =

4.5, 15.5 Hz) 7.29 (1H, t, J = 8.0 Hz), 7.61 (1H, s), 7.78 (2H, br s), 7.89 (1H, d, J = 8.0 Hz), 7.97 (1H, d, J = 8.0 Hz), 11.12 (1H, s). These data match those reported in the literature. Note that the signal due to the α-proton is obscured by a large water peak resulting from HCl added to the solution to improve the solubility of the sample.

3.1.2 Synthesis of 4-nitrotyptamine[3]

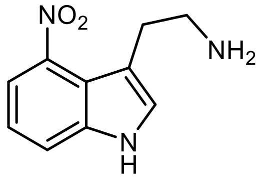

To a stirred solution of 2-cyclohexan-1-one in cyclohexanol (1 % v/v 0.6 mL), 4-nitro-L-tryptophan (41 mg, 0.16 mmol) was added and the resulting suspension was heated to 154 °C under reflux. After 2 hours the reaction was cooled to room temperature and diluted with chloroform (1 mL). The solution was passed through a silica gel pad and washed with water (2 x 2 mL). The organic layer was concentrated *in vacuo* and further diluted with methanol (1 mL) before being purified by reversed phase HPLC (see Table S4) to give 4-nitrotryptamine (7 mg, 0.034 mmol, 21 %). ^{1}H NMR (400 MHz, D_2O) δ: 3.19 (m, 2H), 3.26 (m, 2H), 7.32 (t, 1H, J = 8.0 Hz), 7.56 (s, 1H), 7.87 (d, 1H, J = 8.0 Hz), 8.01 (d, 1H, J = 8.0 Hz). ^{13}C NMR (125 MHz, D_2O) δ: 25.18, 40.85, 108.46, 118.10, 118.47, 119.32, 121.77, 130.37, 139.38, 141.39. *m/z* (ESI$^+$): calculated for $[C_{10}H_{12}N_3O_2]^+$: 206.0924, found: 206.0924

3.2 Synthesis of 2-(benzo[b]thiophen-3-yl)ethanamine[3, 4]

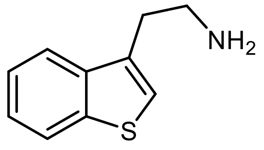

To a stirred solution of 2-cyclohexan-1-one in cyclohexanol (1% v/v, 0.6 mL), 3-(benzothien-3-yl)-D-alanine (90 mg, 0.41 mmol) was added and the resulting suspension was heated to 154 °C under reflux. After 2 h, the reaction was cooled to room temperature and diluted with chloroform (1 mL). The dark brown solution was passed through a silica gel pad and then washed with water (2 x 2 mL). The organic layer was concentrated *in vacuo* to give the desired product as a pale brown solid (22.6 mg, 0.13 mmol, 32 %).

^{1}H NMR (400 MHz, MeOD) δ: 2.96 (s, 2H), 3.23 (s, 2H), 7.22 (s, 1H), 7.29 (m, 2H), 7.74 (s, 1H), 7.79 (d, 1H). *m/z* (ESI$^+$): calculated $[M+H]^+$: 178.0690 Found: 178.0689.

4. Supplementary figures

*Nde*I

CATATGGTGACCGTGCCGAGCCCGCTGGCGGACCCGAGCATTGTGCCGGACCCGTACCCGGTTTACGCGGACCTGGCGC
AACGTCGCCCGGTTCACTGGGTTGAACGTCTGAACGCGTGGGCGGTGCTGACCTACGCGGATTGCGCGGCGGGTCTGAA
GGACCCGCGTCTGACCGCGGACCGTGGTACCGAGGTTCTGGCGGCGAAATTCCCGGGTCAGCCGCTGCCGCCGGACAAC
ATCTTTCACCGTTGGACCAAGAACGTGGTTATGTATACCGATCCGCCGCTGCATGATGCGCTGCGTCGTAGCGTGCGTGC
GGGTTTCACCCGTGCGGCGCACCAACACTATGATCAGGTGCTGCAAAAAGTTGCGCATGACCTGGTTGCGAGCATCCCGG
CGGGTGCGACCGAAATTGATGCGGTGCCGGCGCTGGCGGCGGAGCTGCCGGTGCGTAGCGCGGTTCATGCGTTCGGTG
TTCCGGAGGAAGACCTGGGCTTTCTGATCCCGCGTGTGAACACCATTATGACCTACCACAGCGGTCCGAAGGATCAGCCG
GTTACCCAAGAAATCATTCTGGAGAAACTGACCGACCTGCACACCTATGCGAGCGAGCTGCTGCAGGGCATGCGTGGCA
AGGTGCTGCCGGATACCGTTATTGCGCGTCTGGCGGCGGCGCAAGATGGTCTGACCGAAACCACCCCGGAGCAGACCGT
TCACCAACTGGCGCTGGTGTTCATTGCGCTGTTTGCGCCGACCACCCCGGGTAGCCTGAGCAGCGGTACCCTGGCGTTTG
CGCGTAACCCGCGTCAGGTGGAACGTTTTCTGGCGGATCAAGCGTGCGTTGACAACACCGCGAACGAGGTGCTGCGTTA
CAACGCGAGCAACCAGTTCACCTGGCGTGTGGCGGCGAAAGATGTTGAAATGGGTGGCGTGCGTATCGAGGCGGGTCA
AACCCTGGCGCTGTTTCTGGGCAGCGCGAACCGTGACGCGAACATGTTCGAACGTCCGAACGACTTTGATCTGGACCGTC
CGAACAGCGCGCGTCACCTGAGCTTCGGTCAGGGCGTTCATGCGTGCCTGGCGGCGCAGCTGATCAGCCTGCAACTGAA
GTGGTTCTATGTGGCGCTGCTGAACCGTTTTCCGGGTATCCGTACCGCGGGCGAGCCGATTTGGAACGAAAACCTGGAGT
TTCGTAGCCTGCGTAGCCTGCCGCTGAGCCTGCGTGCGAAAAGCAAGAAAATCCCGCTGGGTGGCATTCCGAGCCCGAG
CACCGAACAGAGCGCGAAGAAAGTTCGTAAGAAAGCGGAGAACGCGCACAACACCCCGCTGCTGGTGCTGTACGGCAG
CAACATGGGTACCGCGGAAGGTACCGCGCGTGATCTGGCGGACATTGCGATGAGCAAGGGTTTCGCGCCGCAAGTTGCG
ACCCTGGATAGCCATGCGGGTAACCTGCCGCGTGAGGGTGCGGTGCTGATTGTTACCGCGAGCTATAACGGTCACCCGCC
GGACAACGCGAAGCAGTTTGTGGATTGGCTGGACCAAGCGAGCGCGGATGAAGTGAAAGGCGTTCGTTACAGCGTTTTC
GGTTGCGGCGACAAGAACTGGGCGACCACCTATCAGAAAGTGCCGGCGTTTATCGATGAAACCCTGGCGGCGAAGGGTG
CGGAAAACATTGCGGATCGTGGCGAGGCGGATGCGAGCGACGATTTCGAAGGTACCTACGAGGAATGGCGTGAGCACA
TGTGGAGCGACGTTGCGGCGTATTTTAACCTGGATATCGAAAACAGCGAGGACAACAAAAGCACCCTGAGCCTGCAGTTT
GTGGATAGCGCGGCGGACATGCCGCTGGCGAAGATGCACGGTGCGTTTAGCACCAACGTGGTTGCGAGCAAAGAACTG
CAACAACCGGGCAGCGCGCGTAGCACCCGTCACCTGGAAATCGAGCTGCCGAAAGAAGCGAGCTACCAAGAGGGTGAT
CACCTGGGCGTTATCCCGCGTAACTATGAAGGTATTGTGAACCGTGTTACCGCGCGTTTTGGCCTGGATGCGAGCCAGCA
AATTCGTCTGGAGGCGGAGGAAGAGAAGCTGGCGCACCTGCCGCTGGCGAAAACCGTGAGCGTTGAAGAGCTGCTGCA
GTACGTTGAGCTGCAAGACCCGGTTACCCGTACCCAACTGCGTGCGATGGCGGCGAAGACCGTTTGCCCGCCGCACAAA
GTGGAACTGGAGGCGCTGCTGGAAAAGCAGGCGTACAAAGAGCAAGTGCTGGCGAAGCGTCTGACCATGCTGGAACTG
CTGGAGAAGTATCCGGCGTGCGAAATGAAATTCAGCGAGTTTATCGCGCTGCTGCCGAGCATTCGTCCGCGTTACTATAG
CATCAGCAGCAGCCCGCGTGTTGACGAAAAGCAGGCGAGCATTACCGTGAGCGTGGTTAGCGGTGAAGCGTGGAGCGG
TTACGGCGAGTATAAAGGCATCGCGAGCAACTACCTGGCGGAACTGCAGGAAGGTGATACCATCACCTGCTTCATTAGCA
CCCCGCAAAGCGAATTTACCCTGCCGAAAGACCCGGAAACCCCGCTGATTATGGTTGGTCCGGGTACCGGTGTGGCGCCG
TTCCGTGGCTTTGTTCAGGCGCGTAAGCAACTGAAAGAACAGGGTCAAAGCCTGGGCGAGGCGCACCTGTACTTCGGTT
GCCGTAGCCCGCACGAGGATTACCTGTATCAGGAAGAGCTGGAAAACGCGCAAAGCGAGGGCATCATTACCCTGCACAC
CGCGTTTAGCCGTATGCCGAACCAGCCGAAGACCTATGTGCAGCACGTTATGGAACAAGATGGTAAGAAACTGATCGAG
CTGCTGGACCAGGGCGCGCACTTCTACATTTGCGGTGATGGTAGCCAAATGGCGCCGGCGGTGGAAGCGACCCTGATGA
AAAGCTATGCGGATGTGCACCAGGTTAGCGAGGCGGACGCGCGTCTGTGGCTGCAACAACTGGAAGAAAAGGGTCGTT
ATGCGAAAGATGTGTGGGCGGGCTAAAAGCTT

*Hin*dIII

Figure S1. Codon optimised DNA sequence for His_6-TxtE-BM3R overproduction in *E. coli*. Bases highlighted in green were used as restriction sites to clone into pET28a(+) to add an *N*-terminal His_6-tag. Bases highlighted in red were deleted using site directed mutagenesis to reduce the size of the linker between TxtE and BMR3 by 15aa.

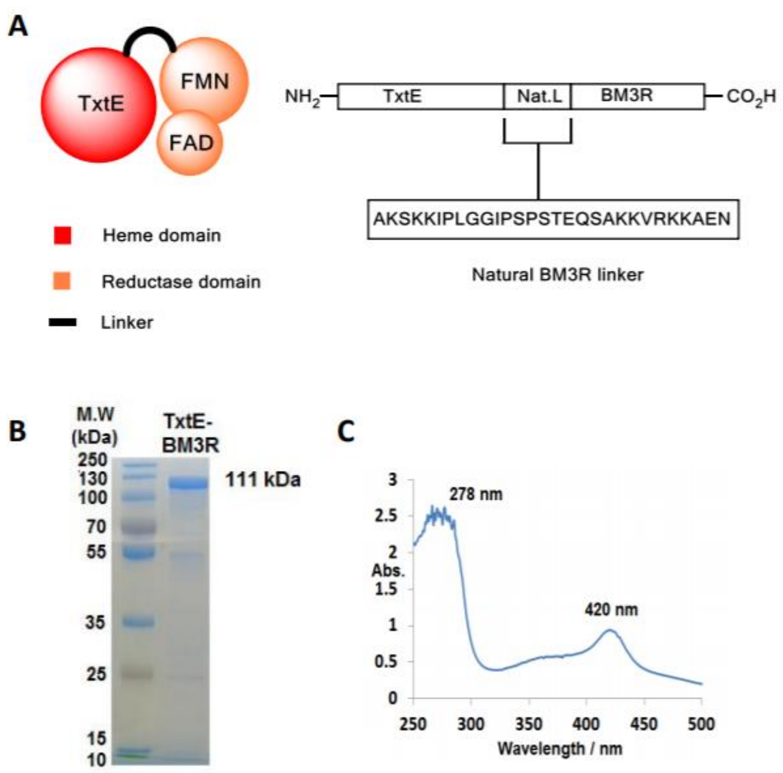

Figure S2. Analysis of TxtE-BM3R. (A) Domain organisation of His_6-TxtE-BM3R and amino acid sequence of the natural heme-reductase linker region. (B) SDS-PAGE analysis of purified His_6-TxtE-BM3R (~111 kDa, right lane). The molecular weight marker is in the left lane. (C) UV-Vis spectroscopic analysis of purified His_6-TxtE-BM3R showing the expected absorbance maximum at 420 nm.

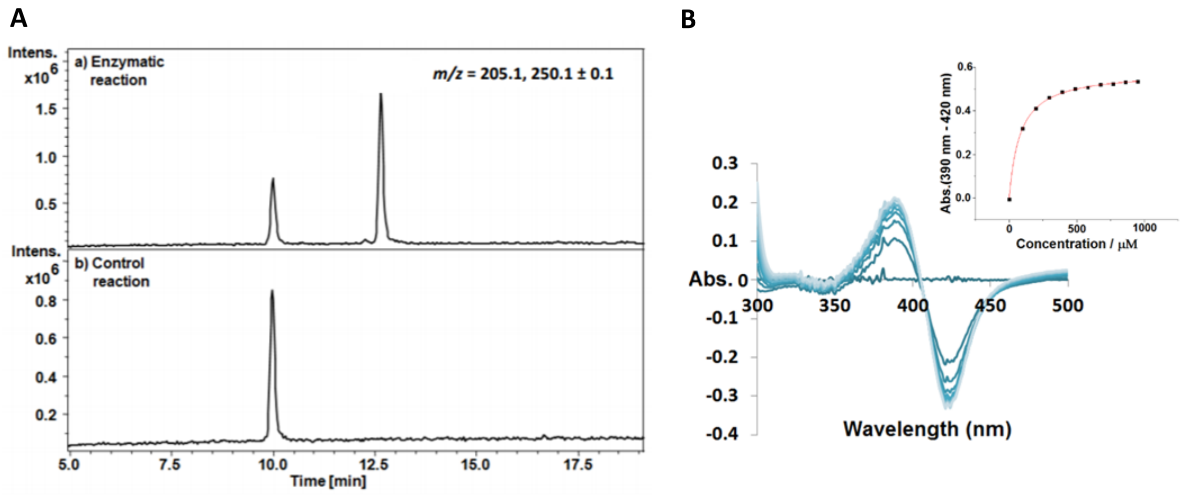

Figure S3. Analysis of L-tryptophan nitration and binding by His_6-TxtE-BM3R. (A) Extracted ion chromatograms at *m/z* = 205.1 and 250.1, corresponding to $[M+H]^+$ for L-tryptophan and 4-nitro-L-tryptophan, respectively, from LC-MS analyses of His_6-TxtE-BM3R incubated with L-tryptophan, NADPH and DEANO (top) and a negative control containing heat-denatured enzyme (bottom). (B) Type I difference spectra from UV-Vis spectroscopic analysis of His_6-TxtE-BM3R titrated with increasing concentrations of L-tryptophan. The inset shows a plot of the difference in absorbance at λ_{max} (390 nm) and λ_{min} (420 nm) versus L-tryptophan concentration. Data are the mean of three measurements and were fitted to the Michaelis-Menten model, giving a K_d of 84 ± 2 µM.

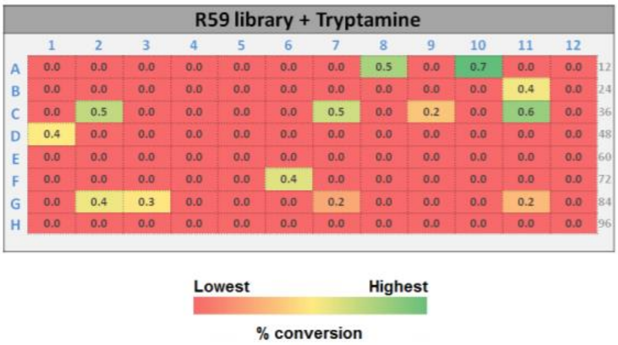

Figure S4. Screening plate heat map for tryptamine nitration by the R59 mutant library of His$_6$-TxtE-BM3R. Reactions were analysed by LC-MS for nitrotryptamine production and conversion was assessed by integrating peaks in UV chromatogams. DNA sequencing revealed that wells A8 and A10 contain an R59C mutant, whereas wells C7 and C11 contain an R59S mutant.

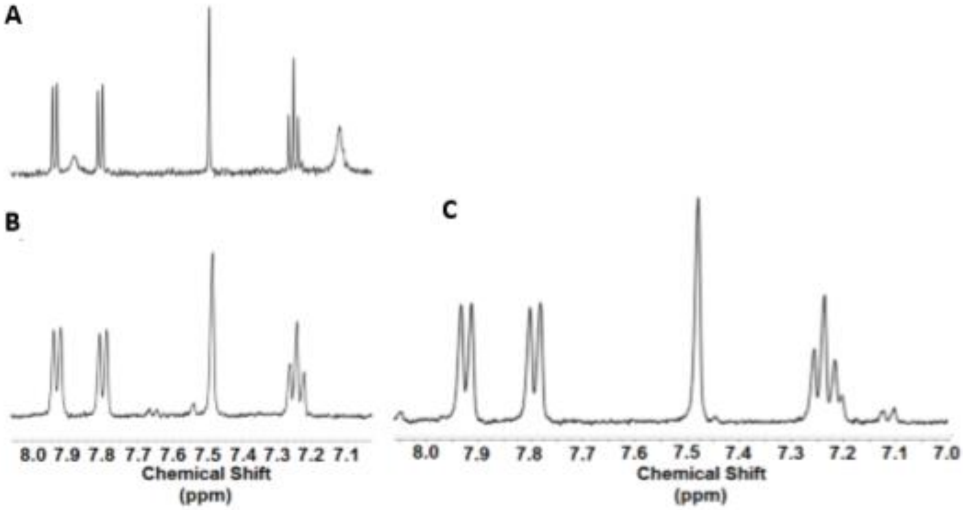

Figure S5. Comparison of the aromatic proton region of ^{1}H NMR spectra of nitrated typtamine produced by His$_6$-TxtE-BM3R(R59C)Δ and a synthetic standard of 4-nitrotryptamine. (A) Product of the enzymatic reaction. (B) Synthetic 4-nitro tryptamine. (C) A mixture of the enzymatic product and synthetic 4-nitrotryptamine.

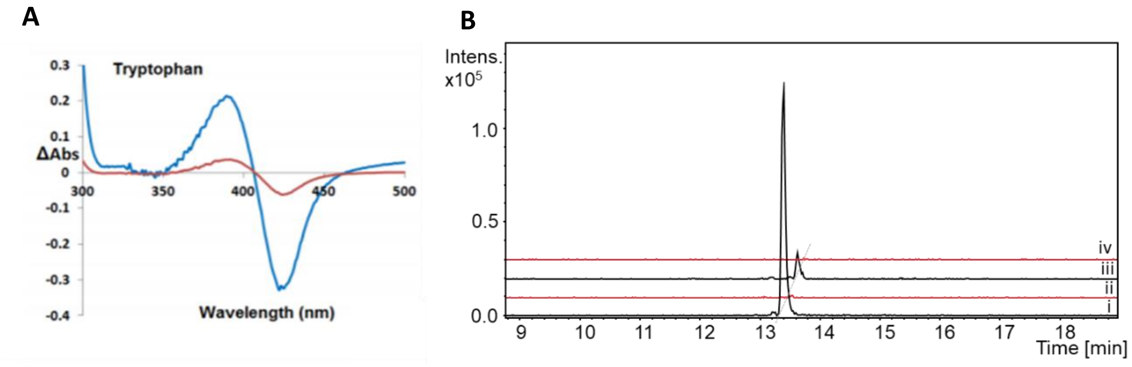

Figure S6. UV-Vis spectroscopic analysis of L-tryptophan binding to unmodified and mutant His_6-TxtE-BM3R and LC-MS analysis of L-tryptophan nitration by unmodified and mutant His_6-TxtE-BM3R (A) UV-Vis difference spectra for a solution of L-tryptophan (0.1 mM) containing 8 µM His_6-TxtE-BM3R (blue) or His_6-TxtE-BM3R(R59C) (red). (B) Extracted ion chromatograms at *m/z* =250.082 ± 0.005, corresponding to $[M+H]^+$ for nitrotryptophan from UHPLC-ESI-Q-TOF-MS analysis of L-tryptophan nitration by His_6-TxtE-BM3R(i) and heat denatured enzyme (ii) or L-tryptophan nitration by His_6-TxtE-BM3R(R59C) (iii) and heat denatured enzyme (iv)

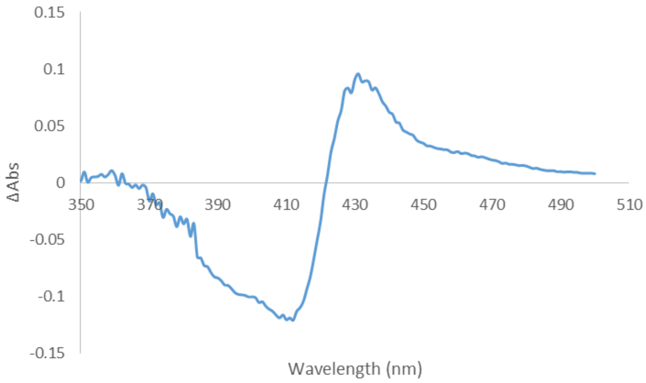

Figure S7. UV-Vis difference spectrum for binding of tryptamine to His_6-TxtE-BM3R(R59C).

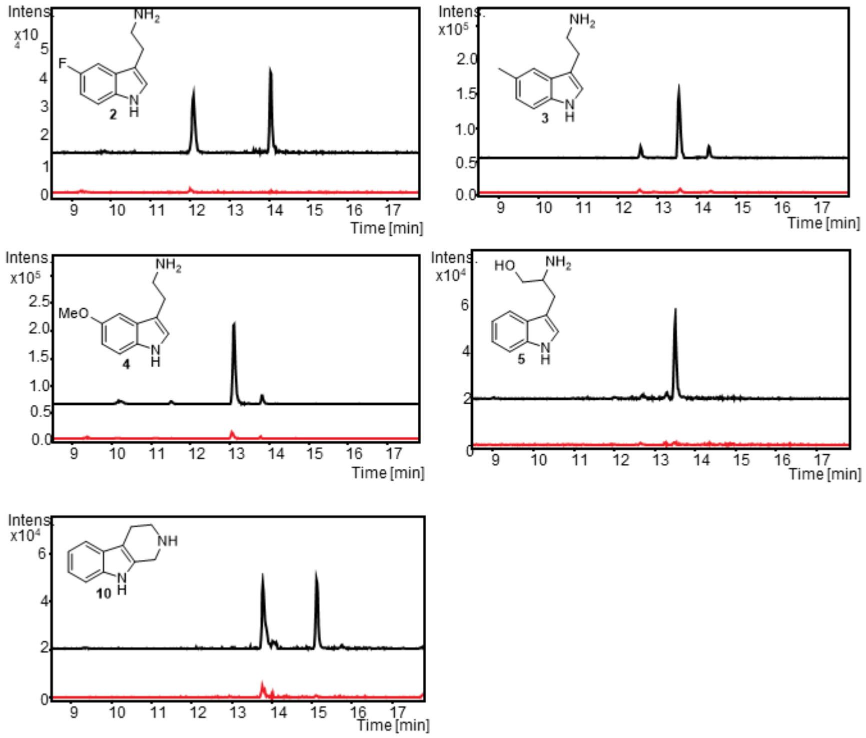

Figure S8. Extracted ion chromatograms at *m/z* =206.924 ± 0.005, 224.083 ± 0.005, 220.1081 ± 0.005, 236.1030 ± 0.005, and 218.0924 ± 0.005, corresponding to $[M+H]^+$ for mono-nitrated **2**, **3**, **4**, **5**, and **11**, respectively, from UHPLC-ESI-Q-ToF-MS analyses of nitration reactions containing **2**, **3**, **4**, **5**, or **11** and His_6-TxtE-BM3R(R59C)Δ (top traces) or heat-denatured enzyme (bottom traces).

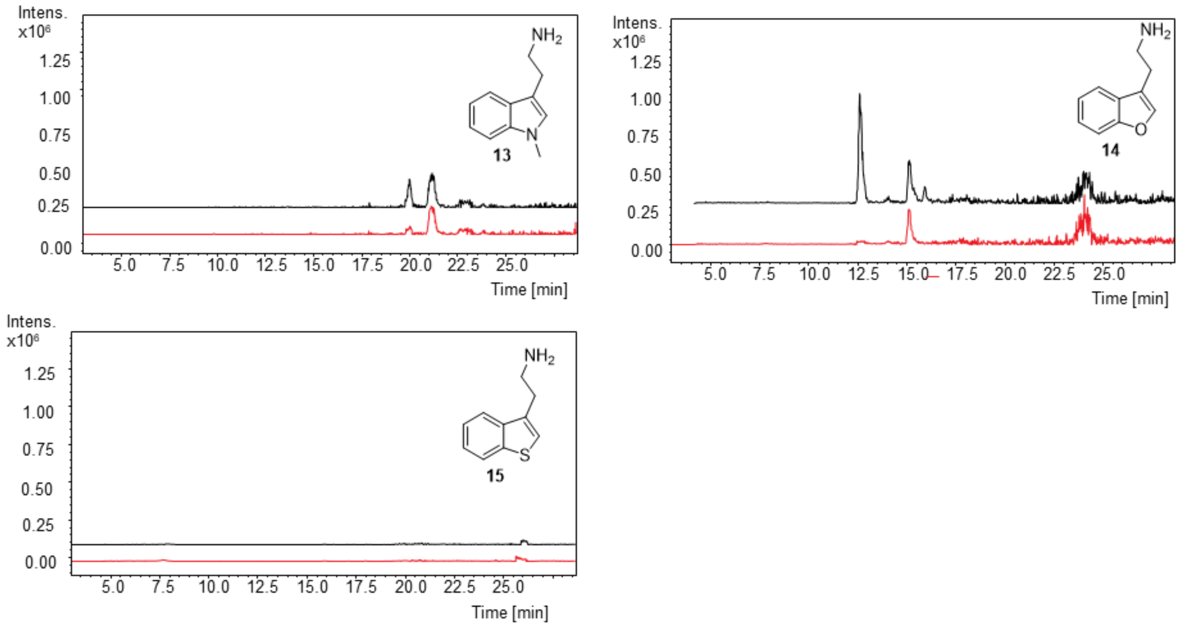

Figure S9. Extracted ion chromatograms at *m/z* =220 ± 0.5, 207 ± 0.5 and 223 ± 0.5, corresponding to $[M+H]^+$ for mono-nitrated **13**, **14** and **15**, respectively, from LC-MS analysis of reaction mixtures containing **13**, **14** or **15** with NADPH, DEANO and TxtEBM3R(R59C) (top traces) or heat-denatured enzyme (bottom traces).

5. Supplementary tables

Table S1*:* Buffers used during protein purification

Process	***Buffer***	***Base***	***NaCl (mM)***	***Imidazole (mM)***	***Glycerol (%)***
Lysis	A	Tris-HCl, pH 8	100	20	0
Elution	B1	Tris-HCl, pH 8	100	50	0
	B2	Tris-HCl, pH 8	100	100	0
	B3	Tris-HCl, pH 8	100	200	0
	B4	Tris-HCl, pH 8	100	300	0
Concentration	C	Tris-HCl, pH 8	100	0	10

Table S2: LC-MS elution conditions for the screening and analysis of the His_6-TxtE-BMR3 R59 mutant library

Time (min)	***% A (Water + 0.1 % formic acid)***	***% B (Methanol + 0.1 % formic acid)***	***Flow rate (mL/min)***
0	100	0	9.000
5	100	0	9.000
34	0	100	9.000
38	0	100	9.000
40	100	0	9.000

Table S3: LC-MS elution conditions for analysis of reactions with TxtE-BM3R_R59C

Time (min)	***% A (Water + 0.1 % formic acid)***	***% B (Methanol + 0.1 % formic acid)***	***Flow rate (mL/min)***
0	100	0	1.000
5	100	0	1.000
30	0	100	1.000
35	0	100	1.000
40	100	0	1.000

Table S4: HPLC elution conditions used for the purification of nitrotryptamine from the scaled-up reaction using purified TxtE-BM3R(R59C)Δ

Time (min)	*% A (Water + 0.1 % formic acid)*	*% B (Methanol + 0.1 % formic acid)*	*Flow rate (mL/min)*
0	100	0	9.000
5	100	0	9.000
34	0	100	9.000
38	0	100	9.000
40	100	0	9.000

Table S5. Comparison of *m/z* observed for products of His$_6$-TxtE-BM3R(R59C)-catalysed nitration of L-tryptophan, tryptamine and tryptamine analogues with calculated values

Substrate	*Molecular formula of nitrated product*	*Calculated* m/z *for [M+H]$^+$ ion*	*Observed* m/z *for nitrated product(s)*
L-tryptophan	$C_{11}H_{12}N_3O_4$	250.0822	250.0813 (t = 13.2 min)
tryptamine (**1**)	$C_{10}H_{12}N_3O_2$	206.0924	206.0916 (t = 9.4 min)<br>206.0919 (t = 13.6 min)
5-fluorotryptamine (**2**)	$C_{10}H_{11}FN_3O_2$	224.0830	224.0819 (t = 12.1 min)<br>224.0823 (t = 14.1 min)
5-methyltryptamine (**3**)	$C_{11}H_{14}N_3O_2$	220.1081	220.1073 (t = 12.6 min)<br>220.1075 (t = 13.6 min)<br>220.1077 (t = 14.3 min)
5-methoxytryptamine (**4**)	$C_{11}H_{14}N_3O_3$	236.1030	236.1029 (t = 13.1 min)<br>236.1024 (t = 13.8 min)
L-tryptophanol (**5**)	$C_{11}H_{14}N_3O_3$	236.1030	236.1021 (t = 13.6 min)
Tryptoline (**10**)	$C_{11}H_{12}N_3O_2$	218.0924	218.0913 (t = 13.8 min)<br>218.0920 (t = 15.1 min)

6. References

[1] S. M. Barry, J. A. Kers, E. G. Johnson, L. Song, P. R. Aston, B. Patel, S. B. Krasnoff, B. R. Crane, D. M. Gibson, R. Loria, G. L. Challis, *Nat. Chem. Biol.* **2012**, *8*, 814–816.

[2] D. K. Romney, D. K. Romney, J. Murciano-Calles, J. E. Wehrmuller, F. H. Arnold, *J. Am. Chem. Soc.* **2017**, *139*, 10769-10776.

[3] H. Mitsunori, E. Yutaka, O. Yasutomo, I.Toahiaki, A. Seiichi, *Chem. Lett.* **1986**, *15*, 893-896.

[4] S. M. N. Efange, D. C. Mash, A.B. Khare, Q. Ouyang, *J. Med. Chem*. **1998**, *41*, 4486-4491
